# Consolidative Radiotherapy for Metastatic Urothelial Bladder Cancer Patients with No Progression and with No More than Five Residual Metastatic Lesions Following First-Line Systemic Therapy: A Retrospective Analysis

**DOI:** 10.3390/cancers15041161

**Published:** 2023-02-11

**Authors:** Amélie Aboudaram, Léonor Chaltiel, Damien Pouessel, Pierre Graff-Cailleaud, Nicolas Benziane-Ouaritini, Paul Sargos, Ulrike Schick, Gilles Créhange, Elizabeth Cohen-Jonathan Moyal, Christine Chevreau, Jonathan Khalifa

**Affiliations:** 1 Department of Radiation Oncology, Institut Claudius Regaud/Institut Universitaire du Cancer de Toulouse—Oncopole, 31000 Toulouse, France; 2 Department of Biostatistics, Institut Claudius Regaud/Institut Universitaire du Cancer de Toulouse—Oncopole, 31059 Toulouse, France; 3 Department of Medical Oncology, Institut Claudius Regaud/Institut Universitaire du Cancer de Toulouse—Oncopole, 31059 Toulouse, France; 4 Department of Radiation Oncology, Institut Curie, 75248 Paris, France; 5 Department of Radiation Oncology, Institut Bergonié, 33000 Bordeaux, France; 6 Department of Radiation Oncology, CHU Brest, 29200 Brest, France

**Keywords:** urothelial bladder cancer, oligometastatic disease, radiotherapy, consolidation, landmark analysis

## Abstract

**Simple Summary:**

Radiotherapy with curative intent in the treatment of metastatic malignancies has shown promising results in several types of primary tumors but experience in bladder cancer is limited and scarce. The objective of our retrospective study was to assess the benefit of consolidative radiotherapy directed to the bladder and to residual metastases among patients with metastatic urothelial bladder cancer and with no progression following systemic therapy. In our analysis, radiotherapy was associated with a valuable and promising survival benefit compared with no local treatment following systemic therapy. The benefit of consolidative radiotherapy needs to be confirmed in prospective clinical trials in the future.

**Abstract:**

Local consolidative radiotherapy in the treatment of metastatic malignancies has shown promising results in several types of tumors. The objective of this study was to assess consolidative radiotherapy to the bladder and to residual metastases in metastatic urothelial bladder cancer with no progression following first-line systemic therapy. Materials/methods: Patients who received first-line therapy for the treatment of metastatic urothelial bladder cancer (mUBC) and who were progression-free following treatment with no more than five residual metastases were retrospectively identified through the database of four Comprehensive Cancer Centers, between January 2005 and December 2018. Among them, patients who received subsequent definitive radiotherapy (of EQD2Gy > 45Gy) to the bladder and residual metastases were included in the consolidative group (irradiated (IR) group), and the other patients were included in the observation group (NIR group). Progression-free survival (PFS) and overall survival (OS) were determined from the start of the first-line chemotherapy using the Kaplan–Meier method. To prevent immortal time bias, a Cox model with time-dependent covariates and 6-month landmark analyses were performed to examine OS and PFS. Results: A total of 91 patients with at least stable disease following first-line therapy and with no more than five residual metastases were analyzed: 51 in the IR group and 40 in the NIR group. Metachronous metastatic disease was more frequent in the NIR group (19% vs. 5%, *p* = 0.02); the median number of metastases in the IR group vs. in the NIR group was 2 (1–9) vs. 3 (1–5) (*p* = 0.04) at metastatic presentation, and 1 (0–5) vs. 2 (0–5) (*p* = 0.18) after completion of chemotherapy (residual lesions), respectively. Two grade 3 toxicities (3.9%) and no grade 4 toxicity were reported in the IR group related to radiotherapy. With a median follow up of 85.9 months (95% IC (36.7; 101.6)), median OS and PFS were 21.7 months (95% IC (17.1; 29.7)) and 11.1 months (95% IC (9.9; 14.1)) for the whole cohort, respectively. In multivariable analysis, consolidative radiotherapy conferred a benefit in both PFS (HR = 0.49, *p* = 0.007) and OS (HR = 0.47, *p* = 0.015) in the whole population; in the landmark analysis at 6 months, radiotherapy was associated with improved OS (HR = 0.48, *p* = 0.026), with a trend for PFS (HR = 0.57, *p* = 0.082). Conclusion: Consolidative radiotherapy for mUBC patients who have not progressed after first-line therapy and with limited residual disease seems to confer both OS and PFS benefits. The role of consolidative radiotherapy in the context of avelumab maintenance should be addressed prospectively.

## 1. Introduction

Bladder cancer is the most common malignancy involving the urinary system, and urothelial (transitional cell) carcinoma is the predominant histological type in the United States and Europe, where it accounts for 90% of all bladder cancers [1]. Among patients with localized muscle-invasive disease, approximately 50% will relapse after radical cystectomy [2,3,4]. Moreover, 10–15% of patients present with unresectable or metastatic disease at diagnosis [5]. A cisplatin-based combination chemotherapy regimen (dose-dense MVAC: methotrexate, vinblastine, doxorubicin, and cisplatin; or GC: gemcitabine and cisplatin) is the preferred initial therapy for patients with metastatic urothelial bladder cancer (mUBC) who are eligible for cisplatin, providing an overall response rate (ORR) of 50–70%, a median overall survival (OS) of 13–15 months and a 5-year OS of approximately 15% [2,6,7,8,9]. Recently, the JAVELIN 100 trial has shown the benefit in OS of maintenance with avelumab in patients whose disease has not progressed following platinum-based chemotherapy (median OS, 21.4 months versus 14.3 months, *p* = 0.001), which now constitutes the standard of care [10].

Oligometastatic disease has emerged as a new paradigm to designate a metastatic state with a limited tumor burden, potentially amenable to cure when local treatments are combined with systemic therapies [11]. While this concept has gained evidence in several malignancies such as non-small cell cancer, colorectal cancer, breast cancer, or prostate cancer [12], the question as to whether the oligometastatic disease concept can be applied to patients with bladder cancer remains uncertain. Recent data suggest that, among patients with metastatic recurrence following radical cystectomy for UBC, a limited number of lesions (≤3) and a limited number of involved organs are independently correlated with cancer-specific survival [13], which argues in favor of local ablative treatment for well-selected patients with oligometastatic UBC.

Local ablative treatment for mUBC can be directed to both the bladder and the distant metastases. In the former case, beyond potential benefit to prevent hematuria or sepsis from primary tumor progression, recent evidence has suggested an oncological benefit of aggressive local treatment of the bladder [14,15]. In the latter case, several series have suggested the benefit of post-chemotherapy retroperitoneal lymph-node dissection among UBC patients with subdiaphragmatic nodal disease, and at least stable disease following chemotherapy resulted in durable long-term survival [16,17,18], with a 5-year OS of 49% in an Italian series [17]. Among patients with various anatomic sites of metastases, the 5-year median OS from metastasectomy ranged from 28% to 72% [19,20,21,22,23,24,25,26,27,28,29], and evidence of a benefit from metastasectomy among mUBC patients with peri-operative chemotherapy has been suggested in a recent meta-analysis [30]. However, in these reports, there was variability in the number of patients who received chemotherapy either before or after metastasectomy, and in the response to chemotherapy prior to metastasectomy, with inclusion of patients who were refractory to chemotherapy in one study [23]. Experience in radiotherapy for the metastases-directed therapy of mUBC is limited and scarce as it relies on heterogeneous populations of oligometastatic UBC and on both palliative and ablative radiation doses [31,32,33,34,35,36,37]. This strategy warrants further investigation, given that it is non-invasive compared with surgery. One way to select among oligometastatic mUBC patients those who would most benefit from local ablative treatment is to propose such a strategy to patients with oligopersistent cancer induced by systemic treatment [38].

The objective of this retrospective study was to assess the benefit of local consolidative radiotherapy (LCR) directed to the bladder and to residual metastases among patients with metastatic urothelial bladder cancer and with no progression following first-line systemic therapy.

## 2. Materials and Methods

### 2.1. Patients

This was a multicenter retrospective study involving four cancer centers affiliated to the Groupe d’Etude des Tumeurs Uro-Génitales (GETUG). Patients were identified through the electronic database of each center between January 2011 and December 2018. Inclusion criteria were as follows: histologically proven metastatic urothelial bladder cancer (mUBC), either synchronous or metachronous, with chemotherapy and/or immunotherapy as the first line of treatment, with stable disease or objective response following treatment according to RECIST 1.1 criteria, with no more than five residual metastatic lesions based on a CT-scan or a ^18^FDG PET-CT, with (in the whole centers) or without (only in Toulouse Cancer Institute) local consolidative radiotherapy (LCR) to the primary tumor for synchronous setting and/or to the residual metastatic lesions.

The patients were then divided into two groups: those who received a LCR (irradiated (IR) group), and those who did not, which constituted the control group (NIR group).

We collected data on patients’ characteristics at diagnosis; disease characteristics including histological variants, timing of metastatic presentation (synchronous vs. metachronous), and number/location of metastases at presentation; previous treatment including first-line systemic treatment and local treatment for patients in a metachronous setting; a radiotherapy schedule in the IR group; and clinical outcome following first-line systemic therapy +/− irradiation.

Our study was approved by our local ethics committee and authorized by the CNIL (Commission nationale informatique et liberté), authorization no. 919375; CRC IUCT-O 19 URO 15.

### 2.2. Counting Metastatic Lesions

The metastasis count was based on the CT scan performed after first-line systemic treatment.

In case of distant lymph node involvement (above the aortic bifurcation), para-aortic lymph node involvement was counted as a single metastatic site regardless of the number of nodes involved, whereas other extra-pelvic lymph nodes were counted individually. At least one of the three following radiological criteria defined the suspicious character of an extra-pelvic lymph node: a small axis greater than 1 cm, and/or the presence of central necrosis, and/or contrast after injection of iodinated product.

### 2.3. Local Consolidative Radiotherapy

In the IR group, LCR was defined as an EQD2Gy dose of at least 45 Gy. Radiotherapy data were collected for each irradiated lesion regarding irradiated volume, radiotherapy technique, and dose/fractionation with an equivalent dose in the 2 Gy-fraction using an α/β ratio of 13 Gy for bladder cancer [39].

### 2.4. Follow Up

Standard follow up included physical examination and a body CT scan every 3 months. Disease status was assessed starting from the beginning of first-line systemic treatment, and progression was defined according to the RECIST 1.1 criteria. In the IR group, the post-irradiation response was reported for each irradiated site according to RECIST 1.1 criteria as well.

### 2.5. Toxicity Following Radiotherapy

In the IR group, toxicity according to CTCAE 7.0 criteria was reported for any irradiated lesion, i.e., bladder, pelvic nodes, and metastatic lesions; due to discrepancies in low-grade toxicity reporting in retrospective studies, only grade ≥ 3 toxicities were reported.

### 2.6. Statistical Analysis

Population characteristics were described by the usual statistics: frequencies, percentages, and number of missing data for each modality for qualitative variables; and median, minimum, maximum, and number of missing data for quantitative variables. Comparisons between groups were made using the χ^2^ test or Fisher’s exact test for qualitative variables and by the Kruskal–Wallis test for quantitative variables.

Overall survival was defined as the time from the date of initiation of first-line metastatic therapy to the date of all-cause death or the date of last news (censored data). In the case of death, the cause of death, whether or not related to the cancer pathology, was recorded.

PFS was defined as the time from the start of first-line metastatic therapy to the date of the first event or the date of last news (censored data). Events considered were progression and death from any cause.

Survival rates were estimated by the Kaplan–Meier method and presented with their 95% confidence intervals. Univariable analyses were performed using the logrank test for qualitative data. Associated variables with a *p*-value of less than 15% and clinically relevant in univariable analyses were considered in multivariable analyses, which were performed using the Cox proportional hazards model and presented with the hazard ratio (HR) and 95% confidence interval for each covariate. A Cox model with a time-dependent variable and a landmark analysis at 6 months (on the event-free living population at 6 months) were used to reduce the bias related to the delay between the initiation of the first line of treatment and the RT (immortal time bias), and to assess the effect of RT on OS and PFS.

All analyses were performed with STATA 16 software, and all tests used were 2-sided with a threshold α at 5%.

## 3. Results

### 3.1. Patients

**Figure 1:**
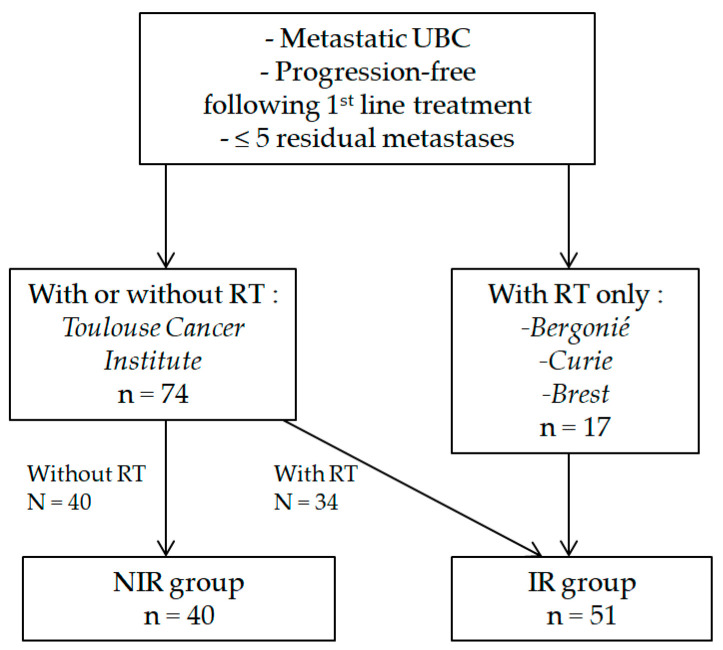
Flow diagram for the selection of patients. Abbreviations: UBC: urothelial bladder cancer; RT: radiotherapy; NIR group: no irradiation group; IR group: irradiation group.

In total, 91 patients were included: 51 in the IR group and 40 in the NIR group. The flow diagram for the selection of patients is presented in Figure 1.

The characteristics of the whole population and of the IR/NIR groups are presented in Table 1. The median age at metastatic presentation was 64 (37–83); ECOG performance status was 0 for 65.9% and 1 for 28.6% of patients, respectively. The distribution of T classification was slightly different between both groups (*p* = 0.019), with more T4 lesions in the IR group (19.1% vs. 5%); in situ carcinoma (CIS) was more frequent in the NIR group (37.9% vs. 12.1%, *p* = 0.018). Metachronous metastatic disease (following definitive treatment of localized UBC) was more frequent in the NIR group (70% vs. 35.3%, *p* = 0.001); the median number of metastases in the IR group vs. in the NIR group was 2 (1–9) vs. 3 (1–5) (*p* = 0.04) at metastatic presentation, respectively. Only one patient (in the IR group) had more than five metastases at presentation (nine lesions). First-line systemic therapy consisted of chemotherapy for all patients except two patients who received durvalumab (in the NIR group) and chemotherapy plus panitumumab (in the IR group), respectively; the median duration of first-line systemic therapy was 3.8 months (0.7–7.4) in the IR group and 3.9 months (1.6–4.9) in the NIR group (*p* = 0.248). Following first-line systemic treatment, a complete response (CR) was found in 27.3% vs. 21.1% in the IR vs. NIR group, and partial response (PR) in 45.5% vs. 42.1% (*p* = 0.289), respectively; the median number of residual lesions after completion of systemic treatment in the IR vs. NIR group was 1 (0–5) vs. 2 (0–5), respectively (*p* = 0.179). Other features were well balanced between both groups.

The landmark analysis was performed on 89 patients with no event (progression or death) at 6 months: 32 patients had radiotherapy within 6 months from the start of systemic therapy (IR group for landmark analysis) and 57 patients had no radiotherapy within 6 months (NIR group for landmark analysis).

### 3.2. Local Consolidative Radiotherapy

In the IR group (n = 51), the median time from the start of systemic treatment to radiotherapy was 5.7 months (1.6–9.6 months), and the median time from the completion of systemic treatment to radiotherapy was 1.9 months (−0.9–5.8 months),

Bladder irradiation was performed in 36 patients (70.6%), of whom 31 also received pelvic lymph node (PLN) irradiation; the main reason for bladder irradiation omission was a history of cystectomy (12/15). The median total dose to the bladder was 64 Gy (45–66 Gy), and the median number of fractions was 33 (15–36).

Metastasis irradiation was performed in 38 patients and was directed to a total of 56 metastases; a median of 1 lesion per patient was irradiated (range: 1–4).

The main metastatic irradiated site was extra-pelvic nodes (29/56; 51.8%). The median EQD2Gy of the 56 irradiated lesions was 53 Gy (45–132 Gy), with a median fraction size of 1.9 Gy (1.7–18 Gy).

The main characteristics of radiotherapy are presented in Table 2.

### 3.3. Treatment Response Analysis

In the IR group, complete response (CR), partial response (PR), stable disease (SD) and progressive disease (PD) on irradiated lesions were observed in 46.2% (24/52), 19.2%, 25% and 9.6%, respectively.

Overall, the best RECIST response following first-line systemic treatment +/− local consolidative radiotherapy in the IR vs. NIR group was CR in 47.1% vs. 30%, PR in 23.5% vs. 37.5%, SD in 21.6% vs. 32.5%, and PD in 7.8% vs. 0%, respectively (*p* = 0.067).

Finally, progression occurred in 40 patients in the IR group (78.4%) vs. 37 patients in the NIR group (92.5%) (*p* = 0.099).

### 3.4. PFS

#### 3.4.1. Whole Population

**Figure 2:**
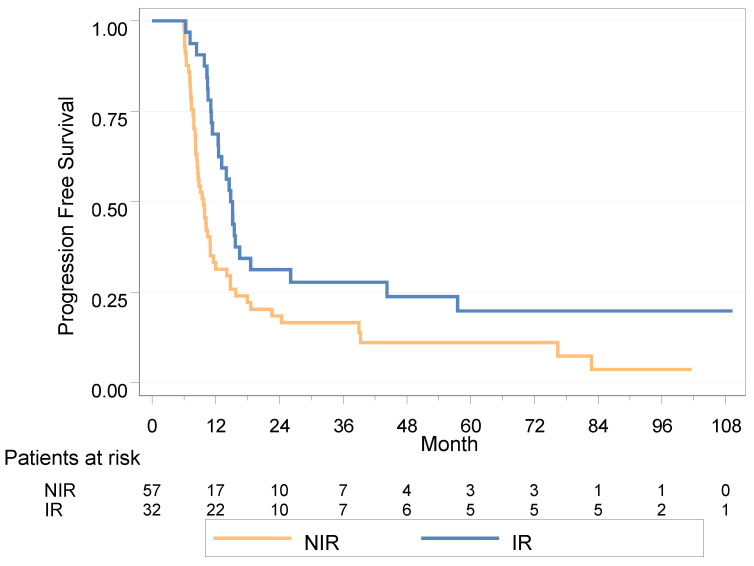
Progression-free survival in landmark analysis at 6 months for NIR (no irradiation) vs. IR group (radiotherapy within 6 months).

After a median follow up of 85.9 months (95% IC (36.7; 101.6)), the median PFS was 11.1 months (95% IC (9.9; 14.1)); using the Cox model with a time-dependent variable, the HR for PFS was 0.45 in the IR group (95% IC (0.28; 0.73), *p* = 0.001) (Figure 2).

In univariable analysis, a delay of 24 months or more between diagnosis and the appearance of metastases appeared deleterious in terms of PFS (HR = 1.80 (1.02–3.20), *p* = 0.040), as did the presence of a liver metastasis (HR = 2.60 (1.23–5.47), *p* = 0.009) (Table S1).

In multivariable analysis, PFS remained improved in the IR group (HR = 0.49 (0.29–0.82), *p* = 0.007), and liver metastasis remained associated with a decrease in PFS (HR = 2.31 (1.07–5.03), *p* = 0.034) (Table S1).

#### 3.4.2. Landmark Population Analysis

In the landmark population analysis at 6 months, the median PFS was 14.8 months in the IR group (11.4; 18.6) versus 9.7 months (8.2; 11) in the NIR group (HR = 0.52 (0.32–0.84), *p* = 0.006); the 2-year PFS was 31.3% vs. 18.5%, respectively.

In univariable analysis, a delay of 24 months or more between diagnosis and the appearance of metastases appeared deleterious in terms of PFS (HR = 1.89 (1.07–3.36), *p* = 0.026), as did the presence of a liver metastasis (HR = 2.76 (1.31–5.81), *p* = 0.005) (Table 3).

In multivariable analysis, none of the variables were statistically associated with PFS; in the IR group there was a trend to improved PFS (HR = 0.57 (0.31–1.07), *p* = 0.082) (Table 3).

### 3.5. OS

#### 3.5.1. Whole Population

**Figure 3:**
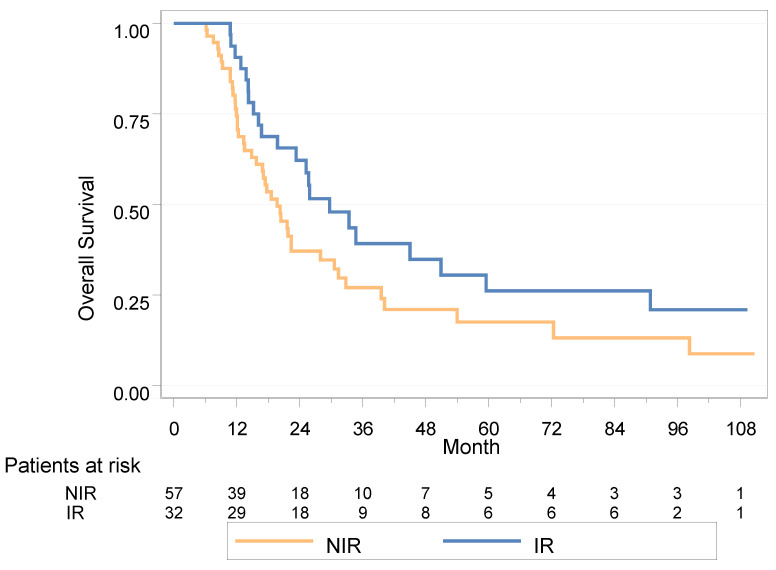
Overall survival in landmark analysis at 6 months for NIR (no irradiation) vs. IR group (radiotherapy within 6 months).

After a median follow up of 85.9 months (95% IC [36.7; 101.6]), the median OS was 21.7 months (95% IC (17.1–29.7)); using the Cox model with a time-dependent, variable the HR for OS was 0.53 in the IR group (95% IC (0.32–0.87), *p* = 0.011) (Figure 3).

In univariable analysis, none of the variables were statistically associated with OS; there was a trend of decreased OS among patients with in situ carcinoma (CIS+) (HR = 1.73 (0.90–3.32), *p* = 0.098) (Table S2).

In multivariable analysis, OS remained improved in the IR group (HR = 0.47, [0.25–0.86], *p* = 0.015) (Table S2).

#### 3.5.2. Landmark Analysis

In the landmark population analysis at 6 months, the median OS was 29.7 months in the IR group (16.7–50.9) versus 19.7 months in the NIR group (14.8–28.0), HR = 0.63 (0.37–1.05), *p* = 0.074; 2-year OS was 62.2% vs. 37.1%, respectively.

In univariable analysis, none of the variables were statistically associated with OS, with a trend for CIS+ patients (HR = 1.80 (0.93–3.47), *p* = 0.076) and number of residual metastases (*p* = 0.12) (Table 4).

In multivariable analysis, OS remained improved in the IR group (HR = 0.48 (0.25–0.92), *p* = 0.026) (Table 4).

### 3.6. Toxicity in the IR Group

Among patients having received pelvic irradiation (bladder and/or PLN irradiation), the rate of grade ≥ 3 toxicity was 2.3% (1/43); it was a grade 3 gastro-intestinal toxicity (proctitis).

Among patients having received metastases-directed irradiation, the rate of grade ≥ 3 toxicity was 2.6% (1/38); it was a grade 3 gastro-intestinal toxicity due to pseudo-occlusive syndrome.

## 4. Discussion

In this retrospective multicentric study, we found that, among patients with at least stable disease and no more than five residual metastases following first-line systemic therapy for metastatic urothelial carcinoma, local consolidative radiotherapy to the bladder (when applicable) and on residual metastases provided significant survival benefit. In multivariable analysis, consolidative radiotherapy conferred a benefit in both PFS (HR = 0.49, *p* = 0.007) and OS (HR = 0.47, *p* = 0.015) in the whole population, and in OS in the landmark analysis at 6 months (HR = 0.48, *p* = 0.026), with only a trend for PFS (HR = 0.57, *p* = 0.082).

Given that radiotherapy in the IR group was performed during the follow up, the landmark analysis at 6 months (from the initiation of systemic therapy) has been realized to preventthe immortal time bias in the IR group [40].The landmark was set at 6 months because most patients in the IR group had received radiotherapy within 6 months of the start of analysis (32/51, 63% of the irradiated patients) and because a landmark beyond 6 months would have substantially decreased the event-free population.

Overall, the main biological rationale to propose local ablative treatment for metastatic malignancies, and especially oligometastatic disease, relies on the seed-and-soil theory. This refers to the secretion of tumor growth factors by the primary tumor, which may promote cancer-cell engraftment in distant organs as they constitute the source of the seed (the circulating-tumor cells), but also may prepare the metastatic soil via the secretion of cytokines that prime the microenvironment in which metastases can potentially develop [41]. In mUBC, data regarding ablative radiotherapy are scare with small series. Overall, in these studies, the 2-year PFS ranged from 19% to 40%, and the 2-year OS ranged from 26% to 75% [31,32,33,34,35,36,37]. However, these data are very heterogeneous in terms of disease characteristics (as high as 35% of oligoprogressive disease in the study by Franzese et al. [36]), radiotherapy schemes (irradiation or not of bladder along with metastases), and timing of radiation delivery compared with systemic therapy (no systemic therapy, upfront radiotherapy with systemic therapy, and systemic therapy followed by consolidative radiotherapy).

In our study, we decided to focus on patients with the oligometastatic state following systemic therapy as a way to select the potentially good candidates for treatment intensification (consolidation approach). We then compared both strategies: local consolidative treatment versus observation. Interestingly, all patients had genuine de novo oligometastatic disease (either synchronous (49.5%) or metachronous (50.5%)), except one patient (in the IR group) who had induced oligopersistent disease (with nine metastatic lesions at diagnosis), according to the ESTRO EORTC oligometastatic classification [38]. This constitutes a major strength of the study as a recent study including any solid malignancy treated with stereotactic radiotherapy to all metastases suggested that, compared with genuine oligometastatic disease, induced oligometastatic disease had a poorer outcome, and that this should be addressed separately in future trials [42,43]. This consolidative approach following systemic therapy has been shown to be promising, especially among patients with non-small-cell lung cancers compared with no local treatment [44,45]. In the largest phase II trial to date, after a median follow up of 38.8 months, there was a benefit from local consolidative therapy in both PFS and OS (median PFS 14.2 months vs. 4.4 months (*p* = 0.022) and median OS 41.2 months vs. 18.9 months (*p* = 0.017)) [44]. Our study, which used a similar design, suggests a similar benefit among mUBC. To the best of our knowledge, this is also the largest cohort of local ablative treatment for oligometastatic bladder cancer.

Moreover, while other series focused either on metastases-directed therapy [34] or on bladder-directed therapy [14,15], our series was innovative as it systematically included patients with local treatment of both primary tumor (except for previous history of cystectomy) and metastases.

On the other hand, our study had some limitations, mainly due to its retrospective nature.

Standards of care for mUBC have recently integrated avelumab maintenance following chemotherapy in non-progressive patients, due to a strong benefit in both PFS and OS in the JAVELIN trial [46]. The patients of our study would have typically been proposed such maintenance according to current guidelines. It is worth mentioning that a 2-year PFS (31%) and a 2-year OS (49%) in the JAVELIN trial compare favorably with our 2-year PFS and 2-year OS in the IR group of the landmark analysis, of respectively 31% and 62%. Whether or not local consolidative radiotherapy can provide similar additional benefit in the context of avelumab maintenance is unknown. In this regard, the ongoing GETUG-AFU V07/BLAD-RAD01 trial (NCT04428554) is a multicentric randomized phase II trial that assesses the benefit of local consolidative radiotherapy to both the bladder and residual metastases among patients without progression following chemotherapy and with no more than three residual lesions (stratified on the type of staging: positron emission tomography (PET) vs. CT only). In this trial, the standard of care with avelumab maintenance following chemotherapy is provided in both arms of treatment, with or without additional radiotherapy.

Finally, radiotherapy towards the tumor has the potency to modify the immune anti-tumor response, as it can induce both immune-stimulatory and immune-suppressive effects [47], and the hypothetical rationale for combining radiotherapy and immunotherapy, especially immune check point inhibitors, is manifold (stimulation of radiation immune-stimulatory effects through in situ vaccination, counteraction of radiation immune-suppressive effects, and immune-resistance tackling) [47,48,49,50]. Therefore, in addition to the mere additive effect of combining both maintenance avelumab and consolidative local radiotherapy, one cannot exclude a synergistic effect of such an association. In this perspective, the optimal fractionation for the irradiation of residual metastases should be determined properly, as the best schedule for maximizing in situ vaccination in combination with immunotherapy may be the delivery of fractions of 8–10 Gy [51,52,53].

## 5. Conclusions

Overall, we found in this retrospective multicentric study that consolidative radiotherapy to the primary tumor and to the residual metastases for mUBC patients who have not progressed after chemotherapy and with limited residual disease seems to confer both OS and PFS benefits. Prospective data in that field with the addition of avelumab are needed.

## Figures and Tables

**Table 1:** Patient characteristics.

	TOTAL N = 91	NIR N = 40	IR N = 51	*p* Value
Center				
Toulouse Cancer Institute	74 (81.3%)	40 (100.0%)	34 (66.7%)	
Institut Bergonié	10 (11.0%)	0	10 (19.6%)	
CHU Brest	3 (3.3%)	0	3 (5.9%)	
Institut Curie	4 (4.4%)	0	4 (7.8%)	
Sex				0.176
Male	81 (89.0%)	38 (95.0%)	43 (84.3%)	
Female	10 (11.0%)	2 (5.0%)	8 (15.7%)	
Age				0.665
Median	62	62	63	
(Range)	(37.0–83.0)	(45.0–83.0)	(37.0–80.0)	
ECOG performance status				0.715
0	60 (65.9%)	27 (67.5%)	33 (64.7%)	
1	26 (28.6%)	10 (25.0%)	16 (31.4%)	
2	4 (4.4%)	2 (5.0%)	2 (3.9%)	
3	1 (1.1%)	1 (2.5%)	0 (0.0%)	
Histological variant				0.526
No	69 (83.1%)	31 (86.1%)	38 (80.9%)	
Yes	14 (16.9%)	5 (13.9%)	9 (19.1%)	
Missing	8	4	4	
Type of variant (n = 14)				0.550
Epidermoid	1 (7.1%)	1 (20.0%)	0 (0.0%)	
Glandular	1 (7.1%)	0 (0.0%)	1 (11.1%)	
Nested	2 (14.3%)	1 (20.0%)	1 (11.1%)	
Micropapillary	5 (35.7%)	1 (20.0%)	4 (44.4%)	
Plasmocytoid	3 (21.4%)	2 (40.0%)	1 (11.1%)	
Sarcomatoid	2 (14.3%)	0 (0.0%)	2 (22.2%)	
Presence of CIS				0.018
No	47 (75.8%)	18 (62.1%)	29 (87.9%)	
Yes	15 (24.2%)	11 (37.9%)	4 (12.1%)	
Missing	29	11	18	
T clinical status at diagnosis				0.019
T1	16 (18.4%)	9 (22.5%)	7 (14.9%)	
T2	52 (59.8%)	22 (55.0%)	30 (63.8%)	
T3	8 (9.2%)	7 (17.5%)	1 (2.1%)	
T4	11 (12.6%)	2 (5.0%)	9 (19.1%)	
Missing	4	0	4	
N clinical status at diagnosis				0.396
N0	46 (51.1%)	23 (57.5%)	23 (46.0%)	
N1	10 (11.1%)	3 (7.5%)	7 (14.0%)	
N2	15 (16.7%)	8 (20.0%)	7 (14.0%)	
N3	19 (21.1%)	6 (15.0%)	13 (26.0%)	
Missing	1	0	1	
Metastatic presentation				0.001
Metachronous	46 (50.5%)	28 (70.0%)	18 (35.3%)	
Synchronous	45 (49.5%)	12 (30.0%)	33 (64.7%)	
Time from diagnosis to metastasis (months)				0.001
Median	0.5	13.1	0.0	
(Range)	(0.0–50.7)	(0.0–250.7)	(0.0–92.6)	
Number of metastatic lesions at metastatic presentation				0.040
Median	2	3	2	
(Range)	(1–9)	(1–5)	(1–9)	
Topography of metastatic lesions at metastatic presentation				
Bone				0.428
No	62 (68.1%)	29 (72.5%)	33 (64.7%)	
Yes	29 (31.9%)	11 (27.5%)	18 (35.3%)	
Lung				0.062
No	73 (81.1%)	29 (72.5%)	44 (88.0%)	
Yes	17 (18.9%)	11 (27.5%)	6 (12.0%)	
Missing	1	0	1	
Liver				0.293
No	83 (91.2%)	35 (87.5%)	48 (94.1%)	
Yes	8 (8.8%)	5 (12.5%)	3 (5.9%)	
Central Nervous System				0.440
No	90 (98.9%)	39 (97.5%)	51 (100.0%)	
Yes	61 (67.0%)	29 (72.5%)	32 (62.7%)	
Extra Pelvic Node				0.326
No	30 (33.0%)	11 (27.5%)	19 (37.3%)	
Yes	61 (67.0%)	29 (72.5%)	32 (62.7%)	
Duration of first-line systemic therapy				0.248
Median	3.8	3.9	3.8	
Range	0.7–7.4	1.6–4.9	0.7–7.4	
Number of residual metastatic lesions following first-line therapy				0.179
Median	1	2	1	
(range)	(0–5)	(0–5)	(0–5)	

Abbreviations: NIR (no irradiation); IR group (radiotherapy).

**Table 2:** Details of local consolidative radiotherapy in the IR group (n = 51).

Irradiated Volume	N (%)	Total Dose Median (Range)	Dose per Fraction (Gy) Median (Range)	Number of Fractions Median (Range)	Total Dose EQD2 Gy
Bladder	36 (70.6%)	64.0 (45.0–66.0)	2.0 (1.7–3.0)	33 (15–36)	64.0 (48.0–66.0)
Pelvic Nodes	39 * (76.5%)	50.0 (24.0–63.0)	1.85 (1.6–3.0)	25 (10–34)	48.5 (24.0–63.5)
Metastasis	38 (74.5%) (56 lesions)	54.0 (30.0–67.5)	1.9 (1.7–18.0)	30 (3–34)	53.0 (45.0–132)

* Of whom eight patients had pelvic lymph node irradiation only without bladder irradiation.

**Table 3:** Univariable and multivariable analysis for progression-free survival (landmark population analysis at 6 months).

Variable		Univariable		Multivariable	
		HR (95% CI)	*p*-Value	HR (95% CI)	*p*-Value
Sex	Male	1			
	Female	0.52 (0.22–1.21)	0.123		
Presence of histological variant	No	1			
	Yes	1.05 (0.55–2.00)	0.881		
Presence of CIS	No	1		1	
	Yes	1.59 (0.86–2.96)	0.137	1.48 (0.79–2.80)	0.224
T clinical status at diagnosis	T1–T2	1			
	T3–T4	1.08 (0.62–1.88)	0.787		
N clinical status at diagnosis	N0	1			
	N+	0.97 (0.61–1.53)	0.891		
Metastatic presentation	Synchronous	1			
	Metachronous	1.29 (0.82–2.02)	0.271		
Time from diagnosis to metastasis	<24 months	1		1	
	≥24 months	1.89 (1.07–3.36)	0.026	2.01 (0.90–4.50)	0.089
Number of metastatic lesions at metastatic presentation	≤3	1			
	>3	1.11 (0.69–1.79)	0.674		
Lung metastatic lesion	No	1			
	Yes	0.98 (0.55–1.72)	0.937		
Liver metastatic lesion	No	1		1	
	Yes	2.76 (1.31–5.81)	0.005	1.60 (0.63–4.07)	0.322
Extra-pelvic node only *	No	1		1	
	Yes	0.65 (0.40–1.07)	0.113	0.56 (0.29–1.11)	0.097
Number of residual metastatic lesions following first-line therapy	0	1			
	1	1.06 (0.56–1.98)			
	≥2	1.38 (0.80–2.36)	0.438		
Local consolidative radiotherapy	No (NIR)	1		1	
	Yes (IR)	0.52 (0.34–0.84)	0.006	0.57 (0.31–1.07)	0.082

* Extra-pelvic nodes are the only sites of metastases. Abbreviations: NIR (no irradiation); IR group (radiotherapy within 6 months).

**Table 4:** Univariable and multivariable analysis for overall survival (landmark analysis at 6 months).

Variable		Univariable		Multivariable	
		HR (95% CI)	*p*-Value	HR (95% CI)	*p*-Value
Sex	Male	1			
	Female	0.55 (0.22–1.36)	0.188		
Presence of histological variant	No	1			
	Yes	1.06 (0.52–2.16)	0.881		
Presence of CIS	No	1		1	
	Yes	1.80 (0.93–3.47)	0.076	1.49 (0.76–2.91)	0.244
T clinical status at diagnosis	T1–T2	1			
	T3–T4	1.12 (0.59–2.01)	0.709		
N clinical status at diagnosis	N0	1			
	N+	1.27 (0.77–2.08)	0.348		
Metastatic presentation	Synchronous	1			
	Metachronous	1.06 (0.65–1.74)	0.805		
Time from diagnosis to metastasis	<24 months	1			
	≥24 months	1.37 (0.76–2.49)	0.293		
Number of metastatic lesions at metastatic presentation	≤3	1			
	>3	1.13 (0.68–1.88)	0.635		
Lung metastatic lesion	No	1			
	Yes	0.94 (0.51–1.74)	0.844		
Liver metastatic lesion	No	1			
	Yes	1.69 (0.76–3.74)	0.192		
Extra-pelvic node only *	No	1			
	Yes	0.73 (0.42–1.26)	0.254		
Number of residual metastatic lesions following first-line therapy	0	1		1	
	1	0.61 (0.29–1.29)		0.68 (0.26–1.80)	
	≥2	1.24 (0.70–2.18)	0.120	1.36 (0.69–2.68)	0.367
Local consolidative radiotherapy	No (NIR)	1		1	
	Yes (IR)	0.63 (0.37–1.05)	0.074	0.48 (0.25–0.92)	0.026

* Extra-pelvic nodes are the only sites of metastases. Abbreviations: NIR (no irradiation); IR group (radiotherapy within 6 months).

## Data Availability

De-identified data from this study are stored by JK.

## Supplementary Materials

The following supporting information can be downloaded at: https://www.mdpi.com/article/10.3390/cancers15041161/s1, Table S1: Univariable and multivariable analysis for progression free survival (whole population, Table S2: Univariable and multivariable analysis for overall survival (whole population).
