# Supplementary material for: Consolidative Radiotherapy for Metastatic Urothelial Bladder Cancer Patients with No Progression and with No More than Five Residual Metastatic Lesions Following First-Line Systemic Therapy: A Retrospective Analysis
Source: Cancers (Basel). 2023 Feb 11;15(4):1161. doi: 10.3390/cancers15041161 (PMC9954747; doi:10.3390/cancers15041161)
Supplement: Supplementary file 1 [file cancers-15-01161-s001.zip › cancers-2142379-supplementary.pdf]

Supplementary Table S1 Univariable and multivariable analysis for progression free survival (whole population)

Variable		Univariable		Multivariable	
		HR (95% CI)	p-value	HR (95% CI)	p-value
Sex	Male	1			
	Female	0.51 (0.22 – 1.18)	0.109		
Presence of histological variant	No	1			
	Yes	1.03 (0.54 – 1.96)	0.927		
Presence of CIS	No	1			
	Yes	1.54 (0.83 – 2.86)	0.167		
T clinical status at diagnosis	T1-T2	1			
	T3-T4	1.04 (0.60 – 1.81)	0.888		
N clinical status at diagnosis	N0	1			
	N+	0.97 (0.62 – 1.52)	0.896		
Metastatic presentation	Synchronous	1			
	Metachronous	1.28 (0.82 – 2.00)	0.277		
Time from diagnosis to metastasis	< 24 months	1		1	
	≥ 24 months	1.80 (1.02 – 3.20)	0.040	1.24 (0.67 – 2.31)	0.49
Number of metastatic lesions at metastatic presentation	≤ 3	1			
	> 3	1.06 (0.66 – 1.71)	0.797		
Lung metastastic lesion	No	1			
	Yes	0.95 (0.54 – 1.67)	0.852		
Liver metastatic lesion	No	1		1	
	Yes	2.60 (1.23 – 5.47)	0.009	2.31 (1.07 – 5.03)	0.034
Extra-pelvic node only *	No	1		1	
	Yes	0.68 (0.41 – 1.10)	0.113	0.81 (0.49 – 1.35)	0.422
Number of residual metastatic lesions following first-line therapy	0	1			
	1	1.11 (0.60 – 2.08)			
	≥ 2	1.41 (0.82 – 2.40)	0.420		
Local consolidative radiotherapy	No (NIR)	1		1	
	Yes (IR)	0.45 (0.28 – 0.73)	0.001	0.49 (0.29 – 0.82)	0.007

* extra-pelvic nodes are the only sites of metastases. Abbreviations : NIR (no irradiation) ; IR (radiotherapy).

Supplementary Table S2 Univariable and multivariable analysis for overall survival (whole population)

Variable		Univariable		Multivariable	
		HR (95% CI)	p-value	HR (95% CI)	p-value
Sex	Male	1			
	Female	0.53 (0.21 – 1.33)	0.171		
Presence of histological variant	No	1			
	Yes	1.06 (0.52 – 2.16)	0.881		
Presence of CIS	No	1		1	
	Yes	1.73 (0.90 – 3.32)	0.098	1.49 (0.76 – 2.91)	0.244
T clinical status at diagnosis	T1-T2	1			
	T3-T4	1.09 (0.59 – 2.01)	0.784		
N clinical status at diagnosis	N0	1			
	N+	1.25 (0.76 – 2.03)	0.377		
Metastatic presentation	Synchronous	1			
	Metachronous	1.08 (0.66 – 1.75)	0.767		
Time from diagnosis to metastasis	< 24 months	1			
	≥ 24 months	1.33 (0.74 – 2.40)	0.342		
	≤ 3	1			

Number of metastatic lesions at metastatic presentation	> 3	1.09 (0.66 – 1.80)	0.744		
Lung metastastic lesion	No	1			
	Yes	0.91 (0.50 – 1.68)	0.771		
Liver metastatic lesion	No	1			
	Yes	1.63 (0.74 – 3.59)	0.233		
Extra-pelvic node only *	No	1			
	Yes	0.76 (0.45 – 1.30)	0.320		
Number of residual metastatic lesions following first-line therapy	0	1			
	1	0.67 (0.32 – 1.38)			
	≥ 2	1.25 (0.71 – 2.20)	0.164		
Local consolidative radiotherapy	No (NIR)	1		1	
	Yes (IR)	0.53 (0.32 – 0.87)	0.011	0.47 (0.25 – 0.86)	0.015

* extra-pelvic nodes are the only sites of metastases. Abbreviations : NIR (no irradiation) ; IR (radiotherapy).
